# Supplementary material for: Examining social isolation and loneliness in combination in relation to social support and psychological distress using Canadian Longitudinal Study of Aging (CLSA) data
Source: PLoS One. 2020 Mar 23;15(3):e0230673. doi: 10.1371/journal.pone.0230673 (PMC7089537; doi:10.1371/journal.pone.0230673)
Supplement: S1 Table — (DOCX) [file pone.0230673.s001.docx]

**Supplemental Table 1. Adjusted analyses for middle-aged (age 45-64) versus older individuals (age 65-85)**

|  | **Tangible support** | **Positive interactions** | **Affection** | **Emotional support** | **Desire for more participation (vs. no desire)** | **High psychological distress (vs. low distress)** |
| --- | --- | --- | --- | --- | --- | --- |
|  | **Age 45-64** | | | | | |
| **Social isolation/loneliness groups** |  |  |  |  |  |  |
| Neither isolated nor lonely vs. only isolated | **0.37 (0.02)** | **0.24 (0.02)** | **0.38 (0.02)** | **0.18 (0.02)** | -0.11 (0.05) | -0.13 (0.08) |
| Neither isolated nor lonely vs. only lonely | **0.34 (0.03)** | **0.47 (0.03)** | **0.40 (0.03)** | **0.41 (0.03)** | **-0.82 (0.08)** | **-0.68 (0.10)** |
| Neither isolated nor lonely vs. isolated and lonely | **0.84 (0.05)** | **0.75 (0.04)** | **0.98 (0.05)** | **0.58 (0.04)** | **-0.70 (0.11)** | **-0.65 (0.13)** |
| Only isolated vs. only lonely | -0.03 (0.03) | **0.23 (0.03)** | 0.03 (0.03) | **0.23 (0.03)** | **-0.71 (0.09)** | **-0.55 (0.11)** |
| Only isolated vs. isolated and lonely | **0.47 (0.05)** | **0.51 (0.04)** | **0.61 (0.06)** | **0.40 (0.05)** | **-0.59 (0.11)** | **-0.52 (0.14)** |
| Only lonely vs. isolated and lonely | **0.50 (0.05)** | **0.28 (0.05)** | **0.58 (0.06)** | **0.17 (0.05)** | 0.12 (0.13) | 0.03 (0.15) |
|  | **Age 65-85** | | | | | |
| Neither isolated nor lonely vs. only isolated | **0.43 (0.02)** | **0.30 (0.02)** | **0.45 (0.02)** | **0.24 (0.02)** | -0.07 (0.05) | -0.06 (0.10) |
| Neither isolated nor lonely vs. only lonely | **0.33 (0.03)** | **0.35 (0.03)** | **0.32 (0.03)** | **0.28 (0.03)** | **-0.71 (0.08)** | **-0.65 (0.12)** |
| Neither isolated nor lonely vs. isolated and lonely | **0.91 (0.05)** | **0.72 (0.04)** | **0.92 (0.05)** | **0.62 (0.09)** | **-0.71 (0.10)** | **-0.66 (0.15)** |
| Only isolated vs. only lonely | -0.10 (0.04) | 0.05 (0.03) | **-0.12 (0.04)** | 0.04 (0.03) | **-0.64 (0.09)** | **-0.59 (0.14)** |
| Only isolated vs. isolated and lonely | **0.48 (0.05)** | **0.41 (0.05)** | **0.47 (0.05)** | **0.38 (0.05)** | **-0.64 (0.11)** | **-0.61 (0.16)** |
| Only lonely vs. isolated and lonely | **0.57 (0.06)** | **0.37 (0.05)** | **0.59 (0.06)** | **0.34 (0.05)** | -0.00 (0.12) | -0.02 (0.17) |

Note: Parameter estimates are shown for least squares mean differences between groups derived from regression analyses (standard errors in brackets). Analyses control for: age group (45-54 vs 55-64/65-74 vs 75-85), sex, education, household income, functional impairment, chronic conditions, depressive symptom, and province of residence at baseline. Statistical significance was assessed using a Bonferroni adjustment, p value of .01/6=.0017. Significant results are bolded.
