# Supplementary material for: Examining social isolation and loneliness in combination in relation to social support and psychological distress using Canadian Longitudinal Study of Aging (CLSA) data
Source: PLoS One. 2020 Mar 23;15(3):e0230673. doi: 10.1371/journal.pone.0230673 (PMC7089537; doi:10.1371/journal.pone.0230673)
Supplement: S2 Table — (DOCX) [file pone.0230673.s002.docx]

**Supplemental Table 2. Adjusted analyses for women versus men**

|  | **Tangible support** | **Positive interactions** | **Affection** | **Emotional support** | **Desire for more participation (vs. no desire)** | **High psychological distress (vs. low distress)** |
| --- | --- | --- | --- | --- | --- | --- |
|  | **Women** | | | | | |
| **Social isolation/loneliness groups** |  |  |  |  |  |  |
| Neither isolated nor lonely vs. only isolated | **0.37 (0.02)** | **0.26 (0.02)** | **0.41 (0.02)** | **0.18 (0.02)** | -0.09 (0.05) | -0.02 (0.08) |
| Neither isolated nor lonely vs. only lonely | **0.33 (0.03)** | **0.40 (0.03)** | **0.36 (0.03)** | **0.33 (0.03)** | **-0.78 (0.07)** | **-0.62 (0.09)** |
| Neither isolated nor lonely vs. isolated and lonely | **0.75 (0.05)** | **0.66 (0.04)** | **0.86 (0.05)** | **0.51 (0.04)** | **-0.77 (0.11)** | **-0.66 (0.14)** |
| Only isolated vs. only lonely | -0.03 (0.03) | **0.14 (0.03)** | -0.05 (0.03) | **0.16 (0.03)** | **-0.70 (0.08)** | **-0.61 (0.11)** |
| Only isolated vs. isolated and lonely | **0.38 (0.05)** | **0.40 (0.04)** | **0.45 (0.05)** | **0.34 (0.04)** | **-0.68 (0.11)** | **-0.65 (0.15)** |
| Only lonely vs. isolated and lonely | **0.42 (0.05)** | **0.26 (0.05)** | **0.50 (0.06)** | **0.18 (0.05)** | 0.02 (0.12) | -0.04 (0.15) |
|  | **Men** | | | | | |
| Neither isolated nor lonely vs. only isolated | **0.41 (0.02)** | **0.27 (0.02)** | **0.39 (0.02)** | **0.23 (0.02)** | -0.11 (0.05) | -0.21 (0.10) |
| Neither isolated nor lonely vs. only lonely | **0.34 (0.04)** | **0.47 (0.04)** | **0.41 (0.04)** | **0.43 (0.04)** | **-0.77 (0.09)** | **-0.75 (0.14)** |
| Neither isolated nor lonely vs. isolated and lonely | **0.98 (0.05)** | **0.82 (0.05)** | **1.04 (0.06)** | **0.67 (0.05)** | **-0.65 (0.11)** | **-0.62 (0.16)** |
| Only isolated vs. only lonely | -0.07 (0.04) | **0.21 (0.04)** | 0.02 (0.04) | **0.20 (0.04)** | **-0.66 (0.10)** | **-0.54 (0.15)** |
| Only isolated vs. isolated and lonely | **0.56 (0.06)** | **0.55 (0.05)** | **0.65 (0.06)** | **0.44 (0.05)** | **-0.54 (0.12)** | **-0.41 (0.17)** |
| Only lonely vs. isolated and lonely | **0.64 (0.06)** | **0.34 (0.06)** | **0.63 (0.07)** | **0.24 (0.06)** | 0.12 (0.14) | 0.13 (0.14) |

Note: Parameter estimates are shown for least squares mean differences between groups from regression analyses (standard errors in brackets). Analyses control for: age group, education, household income, functional impairment, chronic conditions, depressive symptom, and province of residence at baseline. Statistical significance was assessed using a Bonferroni adjustment, p value of .01/6=.0017. Significant results are bolded.
