# Supplementary figures and images for: Examining social isolation and loneliness in combination in relation to social support and psychological distress using Canadian Longitudinal Study of Aging (CLSA) data
Source: PLoS One. 2020 Mar 23;15(3):e0230673. doi: 10.1371/journal.pone.0230673 (PMC7089537; doi:10.1371/journal.pone.0230673)

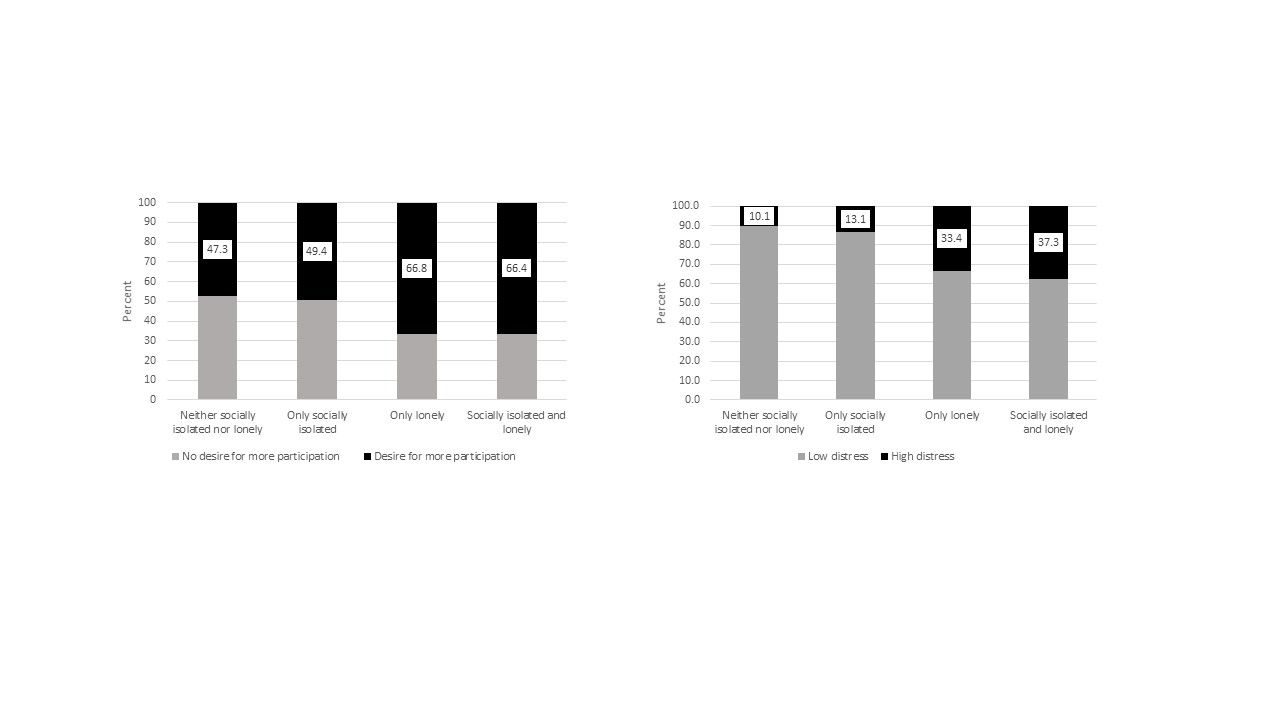

Supplement: S1 Fig — (JPG) [file pone.0230673.s003.jpg]
