# Supplementary material for: Examining social isolation and loneliness in combination in relation to social support and psychological distress using Canadian Longitudinal Study of Aging (CLSA) data
Source: PLoS One. 2020 Mar 23;15(3):e0230673. doi: 10.1371/journal.pone.0230673 (PMC7089537; doi:10.1371/journal.pone.0230673)
Supplement: Attachment — Submitted filename: Response to Reviewers.docx [file pone.0230673.s004.docx]

PONE-D-19-26452
Social isolation and loneliness groups: Associations with social support and psychological distress using Canadian Longitudinal Study of Aging (CLSA) data
PLOS ONE

*Dear Dr. Reppermund;*

*We have revised our manuscript in line with the two Reviewers’ helpful comments. Below please find responses to the Reviewers’ comments. Please note that the page numbers indicated in our response refer to the unmarked manuscript without tracked changes.*

*Thank you for re-considering our paper*.

Journal requirements:

When submitting your revision, we need you to address these additional requirements.

1. Please ensure that your manuscript meets PLOS ONE's style requirements, including those for file naming. The PLOS ONE style templates can be found at
<http://www.journals.plos.org/plosone/s/file?id=wjVg/PLOSOne_formatting_sample_main_body.pdf> and <http://www.journals.plos.org/plosone/s/file?id=ba62/PLOSOne_formatting_sample_title_authors_affiliations.pdf>

[Note: HTML markup is below. Please do not edit.]

Reviewers' comments:

Reviewer's Responses to Questions

**Comments to the Author**

1. Is the manuscript technically sound, and do the data support the conclusions?

The manuscript must describe a technically sound piece of scientific research with data that supports the conclusions. Experiments must have been conducted rigorously, with appropriate controls, replication, and sample sizes. The conclusions must be drawn appropriately based on the data presented.

Reviewer #1: Yes

Reviewer #2: Yes

Reviewer #3: Yes

Reviewer #4: Yes

2. Has the statistical analysis been performed appropriately and rigorously?

Reviewer #1: Yes

Reviewer #2: N/A

Reviewer #3: Yes

Reviewer #4: Yes

3. Have the authors made all data underlying the findings in their manuscript fully available?

The [PLOS Data policy](http://www.plosone.org/static/policies.action#sharing) requires authors to make all data underlying the findings described in their manuscript fully available without restriction, with rare exception (please refer to the Data Availability Statement in the manuscript PDF file). The data should be provided as part of the manuscript or its supporting information, or deposited to a public repository. For example, in addition to summary statistics, the data points behind means, medians and variance measures should be available. If there are restrictions on publicly sharing data—e.g. participant privacy or use of data from a third party—those must be specified.

Reviewer #1: Yes

Reviewer #2: No

*CLSA data are available via application to the Data Access Committee (see* [*https://www.clsa-elcv.ca/data-access*](https://www.clsa-elcv.ca/data-access)*). Data are made available to researchers through a data sharing agreement for the purposes of a specific project and cannot be shared with others.*

Reviewer #3: Yes

Reviewer #4: Yes

4. Is the manuscript presented in an intelligible fashion and written in standard English?

PLOS ONE does not copyedit accepted manuscripts, so the language in submitted articles must be clear, correct, and unambiguous. Any typographical or grammatical errors should be corrected at revision, so please note any specific errors here.

Reviewer #1: No

Reviewer #2: Yes

Reviewer #3: Yes

Reviewer #4: Yes

5. Review Comments to the Author

Please use the space provided to explain your answers to the questions above. You may also include additional comments for the author, including concerns about dual publication, research ethics, or publication ethics. (Please upload your review as an attachment if it exceeds 20,000 characters)

Reviewer #1: This is an interesting analysis that examines the meaning of the potential combinations of presence or absence of loneliness and social isolation in an adult population in Canada.
Specific comments
Title: You may consider deleting the word “groups” from the title, because the meaning may not clear to someone who is only viewing the title. It could mean an intervention group for this population. You mean subtypes rather than groups.

*Good point. We have changed the title of the paper to: “Examining social isolation and loneliness together in relation to social support and psychological distress using Canadian Longitudinal Study of Aging (CLSA) data”*

Abstract. The referral to “loners” or “lonely in a crowd” is not clear to the reader who has not yet read the introduction.

*We are now referring to the four groups more descriptively as ‘socially isolated and lonely’; ‘only socially isolated’; ‘only lonely’; and, ‘neither socially isolated nor lonely’ throughout the paper.*

Page 4. Personally, I do not like the term “lonely in a crowd”. People who have a social network but are lonely are not “in a crowd”, but rather miss a certain intimacy within their social network which may not be that large, but does not qualify for social isolation.

*As noted above, we are no longer using any labels for the four groups.*

Page 5 “the discrepancy theory of loneliness” sounds more like a definition than a theory.

*We have changed this to ‘perspective’, a term that is commonly used in the literature when describing the discrepancy perspective of loneliness (p. 5).*

Page 9, line 171 consider replacing ‘group’ with ‘groups’8

*This typo has been corrected.*

Page 34, in my opinion, the main limitations are not mentioned. Those pertain to
• dichotomizing continuous variables - . social isolation and loneliness are not dichotomous variables and using different cutpoint could change the results.

• Because of the large sample size, effect sizes could be very small despite significant results
• The sample mixes pre-retirement and post-retirement ages despite the differences between those age groups concerning processes concerning loneliness and isolation

*We have added these points in the limitations section (pp. 34/35).*

• The percentage of lonely people in this sample (6.7%+3%=9.7% line 392 on page 20) is much lower than in most other reports of loneliness, especially in the older population.

*The prevalence of loneliness varies widely in the literature, depending on the sample, measure, and cut-off used. Our cut-off is quite stringent; i.e. participants who indicated that they are 3 or more days per week lonely were identified as being lonely. Moreover, our sample included both middle-aged and older adults. Our prevalence estimate of 9.7% lonely is not inconsistent with previous research; e.g., Beutel et al. (2017) found a loneliness prevalence of 10.5% in a sample of 35-74 year olds; Wenger & Burholt (2004), in a sample of older adults, found a prevalence of 9% who were very lonely; and Dykstra (2009) showed a wide range in the prevalence of loneliness across countries: <5% in Denmark; 5-9% in Finland, Germany, Netherlands, and UK; 10-14% in Belgium, France, Ireland, Luxembourg, and Spain; and >19% in Greece and Portugal.*

Reviewer #2: This paper makes a significant contribution of characterizing the “isolated but not lonely” and the “lonely but not isolated” groups on sociodemographic variables and adjustment outcomes.

Introduction
Overall, the arguments can be streamlined by expressing the meaning of certain sentences and paragraphs more clearly.
1. Line 54: Minister “for” loneliness

*This mistake has been corrected.*

2. The sentence from line 61 to 68 is unclear in meaning.

*We have revised this section to make the meaning clearer.*

3. Line 71 to 11: it unclear whether the “on the one hand… on the other hand…” comparison meant to characterize the distinction between social isolation and loneliness only, or also between these two terms and social support. Hence the sentence need to be written to improve its meaning.

*We have revised this sentence.*

4. The paragraph from 152 to 161 can be written to express its meaning more clearly. The sentence from line 154 to 156 is particularly unclear.
*We have revised this section.*

Methods
5. The way in which participants have been split into four groups can be prone to type 1 error. Do the results hold if the analyses performed were continuous instead of categorical?

*We did not conduct analyses with social isolation and loneliness treated as continuous measures for two main reasons: 1) As noted in the paper (p. 8), social isolation and loneliness are often used as dichotomous variables. This approach, as opposed to keeping then continuous variables, is particularly useful as it allows identifying at-risk individuals who could, potentially benefit from interventions. 2) Creating four groups by combining social isolation and loneliness follows the same logic; i.e. it allows identifying specific groups of individuals who may be at risk. We have now revised the introduction to make this rationale clearer (pp. 8/9).*

*In terms of type 1 errors, we certainly agree with the Reviewer. This is why we used a conservative Bonferroni adjustment to test for significant differences between the four groups for each of the outcome measures, with each test evaluated at an alpha of .01/6=.0017. That is, our family-wise error rate is controlled at alpha of 01. Given the conservative nature of the Bonferroni adjustment, it is possible that we therefore committed type 2 errors.*

6. The authors mentioned that other similar studies have used cluster analysis. Have the authors tried cluster analysis on this dataset to see whether the four groups emerged?

*We have at this point not conducted cluster analysis to determine whether the four groups emerged, but simply used an a priori approach to create the four groups we had discussed in a previous, conceptual paper (Newall & Menec, 2018). We recognize that there are many other studies that could be conducted to further explore the four groups. We have added the idea of conducting cluster analysis in the Discussion section (p. 35).*

7. Are the variables marital status and living arrangements (e.g. living alone vs with others) available in this dataset? These variables are quite important for studies on social isolation and loneliness.

*Yes, these variables are in the dataset and were used to create the social isolation variable. See p. 14.*

Results
8. Could graphs be included for all outcome measures?
*We have added graphs for desire for social participation and psychological distress in Supplemental Figure 1.*

Discussion:
9. The take-aways, whether in terms of characterizations of or intervention for each group, needs to be made clearer. Currently, the findings read like lists of observations about each group. It will help the readers digest findings if the observations are organized in a more structured and coherent manner.

*We have revised the Discussion and hope this addresses the Reviewer’s concerns.*

10. Technology-based interventions for the vulnerable group does not sound realistic or sustainable, given that this group has the largest proportion of “low education” and “low income”. Will regular home visits be a more suitable intervention?

*Technology-based interventions do not need to be expensive or difficult – and can be as simple as using the telephone. We have clarified this in the Discussion (p. 31). Home visits are an option, but would be more resource intensive. We have added home visits as another option in the Discussion (p. 31).*

11. Line 534 – 537: “Given that individuals in this group do not have their social needs met, despite having a relatively large social network, they may benefit from developing new friendships.” This sentence does not make sense. Why give people more of what is currently not working for them? It needs to be made clearer how the new friendships are different from existing ones.

*We have revised this sentence to make it clearer that we mean developing new, meaningful social relationships (p. 31).*

12. Line 537: It is curious that the line “As for the vulnerable group…” appeared in the middle of a paragraph ostensibly about the lonely in a crowd group. Is the content in the rest of the paragraph that appears after this line about the vulnerable group or lonely in a crowd group?

*We have revised this sentence (p. 38).*

13. What are the intervention implications of the results from age-based sensitivity analysis for older adults?
*We have added a comment regarding this (p. 34).*

Reviewer #3: It is a great paper about the association of social isolation and loneliness with psychological distress, social support and civic participation. The topic is very interesting and there is a lack in the literature. However some aspects sohould improve:

1.-I think that the introduction should be shorter and focused on the topic (the association between social isolation and loneliness and their effect on civic participation, social support and health)
*In response to the Reviewer’s comment, we have streamlined the introduction.*

2.-It would be more clear to call the groups: 1)Neither lonely nor isolated 2)Only isolated 3)Only lonely 4)lonely and isolated.
*We have adopted the suggested terminology throughout the paper.*

3.-The authors should clarify the statistical analysis. I think that they used chi squared and anova tests for the comparisons in the descriptive analysis, adjusted and unadjusted ordered logistic regresssions models with the social support outcomes and, finally, adjusted and unadjusted logistic regressions models with participation and psychilogical distress as outcome. I also think that they used diferen sratifications of the samle to compare the effect between sepcific groups. But according to the methods section all this things are not clear.
*We have revised the Analytic approach section to clarify the analyses.*

4.-Authors should also clarify why the analysis wit psicological distress as outcome is prospective (like they say in the introduction)
*The reason why psychological distress, but not desire for social participation and social support was analyzed prospectively was simply because of data availability. We have clarified this in the introduction (p. 4).*

5.-In my opnion, the main results are that loneliness but not social isolation showed a significant effect on psychological destress as well as that the effect of loneliness seem to be greater than those of social isolation in most of the outcomes.
*We agree with the Reviewer that loneliness has important health implication, beyond social isolation. We have added a comment to that effect in the Discussion (p. 31).*

6.-The authors should make further cites on rellevant literature such as:
*We have added these citations.*
Holwerda, T. J., Beekman, A. T. F., Deeg, D. J. H., Stek, M. L., van Tilburg, T. G., Visser, P. J., … Schoevers, R. A. (2012). Increased risk of mortality associated with social isolation in older men: only when feeling lonely? Results from the Amsterdam Study of the Elderly (AMSTEL). Psychological Medicine, 42(4), 843–853. <https://doi.org/10.1017/S0033291711001772>

Holwerda, T. J., Deeg, D. J. H., Beekman, A. T. F., van Tilburg, T. G., Stek, M. L., Jonker, C., & Schoevers, R. a. (2014). Feelings of loneliness, but not social isolation, predict dementia onset: results from the Amsterdam Study of the Elderly (AMSTEL). Journal of Neurology, Neurosurgery, and Psychiatry, 85(2), 135–142.

Reviewer #4: Review for PLoS One Journal

Social Isolation and Loneliness Groups: Associations with Social Support and Psychological Distress using Canadian Longitudinal Study of Aging (CLSA) Data

The purpose of this paper was to combine measures of social isolation and loneliness to create different groups and determine their association with social support and psychological distress. The four groups created were the majority group, the loner group, the lonely in a crowd group, and the vulnerable group (individuals in this group are dually socially isolated and lonely). In general, the majority group seemed to have the most positive and beneficial associations with social support and psychological distress.

I applaud the authors of this paper as it is innovative and will contribute to the social isolation and loneliness literature. The authors are correct in that many peer-review manuscripts have not examine social isolation and loneliness together, and this aspect of the manuscript alone greatly contributes to its innovation.

In my review of your manuscript, I have found some critiques which I believe will enhance the quality of the manuscript. The biggest issue in my opinion is regarding the methods. More detailed explanations are necessary for the type of analysis utilized, and also differentiating between the cross-sectional analyses and the prospective analyses.

The critiques of your manuscript organized by each section of your paper, from the abstract to the conclusion. I have included line numbers for where I find these issues.

ABSTRACT:

In the methods subsection of the abstract, it would be helpful to include the type of analyses done in the paper. This includes listing which analyses are prospective and which analyses are cross-sectional, and the type of regression modeling utilized (ordinary least squares, logistic, etc.)
*We have added this information in the abstract.*

INTRODUCTION:

Line 97: This study is actually over 30 years old now, so it should read over 30 years.

*Thanks for catching this mistake. We have revised this accordingly.*

Lines 123-125: The authors raise a very important point here, in that most studies have not examined the interactive effects of social isolation and loneliness on health. That being said, the authors should also consider including studies by Cornwell and Waite (2009) and Coyle and Dugan (2012). These studies have simultaneously examined social isolation and loneliness (or Cornwell and Waite’s notion, social disconnectedness and perceived isolation) on health. Please include these studies when covering the literature.
*We have added these citations.*

Lines 162-167: Steptoe and colleagues (2013) also examined an interaction effect between social isolation and loneliness on mortality, and the interaction (I believe) was found to be non-significant.

*We have added this article. The study did, indeed, not find a significant interaction.*

Lines 260-263: There needs to be substantially more information on the amount of missing here. In particular, which measure had the most missing and what measure had the least missing? Were the missing enough to substantially affect the analysis? In general, if the missing are greater than 10%, this can represent a significant problem in the analysis.

Also, how were missing data handled? (I assume through listwise deletion?) If this is the case, then it should be stated.
*We have added a comment re missing data (which was less than 3%) and also now note that we used list-wise deletion of missing data (p. 19).*

Also, a final sample size should be stated for each of the analyses.
*We have added the unweighted sample size in the tables 3-5.*

METHODS

In lines 354-363, the authors discuss having a baseline mental health variable to control for the effects of mental health at baseline. I agree with the authors and think, overall, it would be better to include psychiatric distress at baseline and as an outcome. That being said, I believe it would be helpful to include (in supplementary analyses or aside from the main analyses) another set of analyses without the baseline mental health variable. It would be helpful to do this to determine the consistency of the results and to determine if any of the relationships change.

*We have conducted a sensitivity analysis that did not include baseline depression in the model. The results remain the same (p. 24).*

In lines 380-385: There needs to be a stronger description of the analytic methods used in these analyses. If these are prospective analyses, then that should be stated. For example, if this is a lagged variable analysis or hierarchical modeling analysis, then this should be stated as well.
*We have revised the methods section to clarify the analyses (p. 18).*

RESULTS

No changes necessary.


DISCUSSION

Overall, the discussion is written very well.

From lines 537 to 543 is somewhat of a weird transition. A few lines above, it starts with the most vulnerable group, and then it transitions to the lonely in a crowd group. From 537-543, it then goes back to the most vulnerable group. I would suggest discussing one group at a time instead of going back and forth between groups to increase clarity.

*We had revised this section (p. 37).*

6. PLOS authors have the option to publish the peer review history of their article ([what does this mean?](https://journals.plos.org/plosone/s/editorial-and-peer-review-process#loc-peer-review-history)). If published, this will include your full peer review and any attached files.


If you choose “no”, your identity will remain anonymous but your review may still be made public.


**Do you want your identity to be public for this peer review?** For information about this choice, including consent withdrawal, please see our [Privacy Policy](https://www.plos.org/privacy-policy).

Reviewer #1: No

Reviewer #2: No

Reviewer #3: No

Reviewer #4: No


[NOTE: If reviewer comments were submitted as an attachment file, they will be attached to this email and accessible via the submission site. Please log into your account, locate the manuscript record, and check for the action link "View Attachments". If this link does not appear, there are no attachment files to be viewed.]


While revising your submission, please upload your figure files to the Preflight Analysis and Conversion Engine (PACE) digital diagnostic tool, <https://pacev2.apexcovantage.com/>. PACE helps ensure that figures meet PLOS requirements. To use PACE, you must first register as a user. Registration is free. Then, login and navigate to the UPLOAD tab, where you will find detailed instructions on how to use the tool. If you encounter any issues or have any questions when using PACE, please email us at [figures@plos.org](mailto:figures@plos.org). Please note that Supporting Information files do not need this step.

*In compliance with data protection regulations, you may request that we remove your personal registration details at any time.* [*(Remove my information/details)*](https://www.editorialmanager.com/pone/login.asp?a=r)*. Please contact the publication office if you have any questions.*
